# A Risk Management Framework for Triadic Telemedicine (RMFTT): A Conceptual Development Study

**DOI:** 10.3390/healthcare14213747

**Published:** 2026-11-03

**Authors:** Alexander Gamus, Gabriel Chodick, Yoram Blachar, Tamar Shalom

**Affiliations:** 1 Faculty of Health Systems Management, Academic Center of Law and Business, Ramat Gan 52520, Israel; chodick@tauex.tau.ac.il (G.C.); yoramblachar@gmail.com (Y.B.); tamar.shalom.100@gmail.com (T.S.); 2 Gray Faculty of Medical & Health Sciences, Tel-Aviv University, Tel Aviv 69978, Israel; 3 Division of Community Medical Services, Clalit Health Services, Tel Aviv 62098, Israel

**Keywords:** triadic telemedicine, assisted telemedicine, risk management, patient safety, telenursing, digital health, conceptual framework

## Abstract

**Background:** The triadic model of assisted telemedicine, in which a trained nurse or caregiver facilitates the clinical encounter between a patient and a remote physician, holds considerable potential for improving access, diagnostic accuracy, and continuity of care. However, the distinctive risk architecture of this three-party model remains poorly characterized. Existing telemedicine risk literature addresses predominantly dyadic consultations, leaving the triadic context without a dedicated, integrative framework. **Objective:** This study aims to address the absence of a dedicated risk management framework for the triadic telemedicine context by proposing the Risk Management Framework for Triadic Telemedicine (RMFTT)—a structured, domain-specific tool for identifying, assessing, mitigating, and monitoring the principal risks inherent to nurse-facilitated assisted telemedicine. **Methods:** This conceptual framework development study employs a narrative literature review of PubMed, Scopus, Web of Science, and Google Scholar publications from January 2015 to June 2026, organized across five risk domains: clinical and diagnostic risk, communication risk, legal and liability risk, data privacy and cybersecurity risk, and workforce competency risk. Extracted evidence is synthesized using a structured risk architecture matrix, distinguishing risk type, source, likelihood, and consequence severity within the triadic telemedicine context. **Results:** The synthesis yields the Risk Management Framework for Triadic Telemedicine (RMFTT), a two-axis conceptual model mapping five risk domains against four risk management stages: identification, assessment, mitigation, and monitoring. Each domain is populated with literature-derived risk indicators and corresponding mitigation strategies tailored to the triadic care setting. **Conclusions:** To our knowledge, no dedicated risk management framework has previously been proposed for the triadic telemedicine context. The RMFTT provides the first structured, domain-specific tool for identifying, assessing, mitigating, and monitoring risk across the five principal dimensions of assisted triadic telemedicine. It offers actionable guidance for clinicians, nurse-facilitators, health system managers, and policymakers, and establishes a foundation for subsequent empirical validation across diverse healthcare settings.

## 1. Introduction

The rapid global expansion of telemedicine, accelerated by the COVID-19 pandemic, has fundamentally reshaped the delivery of healthcare. Telemedicine has evolved from a niche service for geographically isolated populations into a mainstream modality of healthcare delivery, with increasing integration across primary care, specialty services, chronic disease management, mental health, and post-acute follow-up [1,2,3]. Within this expanding landscape, telemedicine has demonstrated meaningful benefits across a broad range of clinical applications while simultaneously introducing new challenges for clinical governance, patient safety, and quality assurance [4].

Yet the speed and scale of this expansion have outpaced the development of structured risk management frameworks. Research gaps remain regarding whether telemedicine can fully replace in-person services without compromising care quality, and existing models and frameworks do not sufficiently incorporate the multidimensional risks crucial in the healthcare context, underscoring the need for a more comprehensive framework [5,6]. While safety concerns in dyadic telemedicine, involving a single patient interacting directly with a remote physician, have begun to attract scholarly attention, the risk architecture of more complex, multi-party configurations remains largely uncharacterized.

Among these configurations, the triadic model of assisted telemedicine presents a particularly distinctive and underexamined risk profile. In this model, a trained nurse or caregiver acts as an on-site facilitator, mediating the clinical encounter between the patient and a remote physician. This arrangement introduces a third actor into the consultation—one who simultaneously handles patient data, performs preliminary clinical assessments, operates connected diagnostic devices, and manages the communication flow between patient and physician. To illustrate: an elderly patient with diabetes in a rural clinic connects remotely with her internist; a trained nurse-facilitator—referred to in the American literature as a “telepresenter” [7]—based on site measures vital signs, operates a digital stethoscope, and ensures the patient’s concerns are clearly communicated, functions that the remote physician cannot perform but depends upon for clinical decision-making. Each of these functions carries its own risk dimension, and their interaction within a single consultation creates a compounded risk architecture that differs fundamentally from either face-to-face care or standard dyadic telemedicine.

Prior work has established the structural foundations and adoption dynamics of the triadic model, as well as its communicative dimensions [8,9]. The present study builds on that foundation by addressing the risk dimension, an aspect that has received no dedicated treatment in the triadic telemedicine literature and that remains underserved in the broader telehealth patient safety field. Despite growing recognition that patient safety is a central challenge of telemedicine expansion, existing risk frameworks have been developed for dyadic configurations and cannot adequately capture the compounded risk architecture introduced by a three-party consultation involving a nurse-facilitator intermediary.

This gap is not merely academic. Barriers to telemedicine implementation, including limited broadband access, digital literacy gaps, reimbursement uncertainties, and workflow misalignments, disproportionately affect rural and underserved populations, raising equity concerns—precisely the populations for whom the triadic facilitated model is most relevant and most frequently deployed. Without a structured risk management approach, the expansion of assisted telemedicine into these settings risks exposing the most vulnerable patients to compounded clinical, communicative, legal, and cybersecurity harms.

The present study addresses this gap by proposing the Risk Management Framework for Triadic Telemedicine (RMFTT)—an integrated, domain-specific risk management framework tailored to the assisted triadic telemedicine context. Drawing on a narrative review of published literature from January 2015 to June 2026, the RMFTT organizes risk across five domains and maps each domain against four risk management stages: identification, assessment, mitigation, and monitoring. The framework is designed to be actionable for clinicians, nurse-facilitators, health system managers, and policymakers, and to serve as a foundation for subsequent empirical validation across diverse healthcare settings.

## 2. Background and Rationale

### 2.1. Risk Management in Healthcare: Conceptual Foundations

Clinical risk management is defined as the systematic process of identifying, assessing, mitigating, and monitoring risks that may cause harm to patients, staff, or organizations within the healthcare environment [10]. Adverse events in healthcare are primarily attributable to system failures rather than individual error, a recognition that has shifted risk management from reactive incident response toward proactive governance frameworks embedded in organizational culture and clinical workflow [11]. The World Health Organization’s 2021–2030 Global Patient Safety Action Plan explicitly frames risk management as a mandatory strategic objective for all health systems [12]. Within this context, effective risk management necessitates not only a systematic identification of hazard sources but also an understanding of how risks interact and propagate across the care chain.

The application of risk management principles to digital health and telemedicine is comparatively recent and remains methodologically immature. A systematic review of risk management and patient safety in digital health found that healthcare systems’ multiple interconnected factors form a dense network whose imbalance can compromise patient safety, and that expansion of proactive risk management programs is urgently needed [13]. Despite this recognition, the literature on telemedicine risk management remains fragmented, tending to examine individual risk categories in isolation rather than integrating them into coherent, actionable frameworks applicable to specific telemedicine configurations.

### 2.2. The Telemedicine Risk Landscape: Scope and Limitations of Current Frameworks

A systematic review and meta-analysis covering telemedicine adoption between 2020 and 2025 identified that implementation challenges and the impact on clinical outcomes, access, and quality remain under investigation, with barriers including digital literacy gaps, reimbursement uncertainties, and workflow misalignments disproportionately affecting underserved populations [1]. Patient safety risks in telehealth are systemic, affecting not only healthcare professionals but also the environment and organizational context, and despite its central relevance, patient safety remains an underexplored aspect of telehealth with notable gaps in regulatory frameworks and a lack of comprehensive monitoring guidelines [14].

Several risk frameworks have been proposed for telemedicine and e-health more broadly. A comprehensive risk management framework for e-health care delivery proposed a systematic process covering risk identification, assessment, mitigation, and continuous review, but identified that current frameworks fail to adequately address the specific risks of e-health—highlighting the urgent need for a more specialized approach [5]. An emergent research and policy framework for telehealth encompassed regulatory, delivery system, and outcome dimensions, but did not address multi-party configurations [6]. Yet none of these frameworks has been developed for multi-party telemedicine configurations, and none addresses the risk architecture introduced by a professional intermediary operating between the patient and the remote physician.

### 2.3. The Triadic Model and Its Distinctive Risk Architecture

The triadic model of assisted telemedicine represents a structurally distinct configuration in which a trained nurse or caregiver acts as an on-site facilitator, mediating the clinical encounter between the patient and the remote physician. In the United States, this facilitating role is commonly referred to as the “telepresenter” or “patient-site presenter”—a trained professional who serves as the arms and hands of the remote physician, performing physical assessments, operating diagnostic peripherals, and managing the communication flow of the encounter [7]. Throughout this paper, the primary term used is “nurse-facilitator,” while acknowledging that the RMFTT applies across the full spectrum of facilitator types, with risk profiles calibrated accordingly. This configuration addresses a fundamental limitation of self-administered telemedicine by extending reach to patients with mobility, cognitive, or technological constraints through point-of-care diagnostics and guided self-care. The structural and communicative dimensions of this model have been established in prior work [8,9]. Real-world implementation experience from nurse-facilitated telemedicine programs further supports the clinical and operational feasibility of assisted telemedicine models in primary care and chronic disease management settings [14].

However, the facilitator role simultaneously generates a distinctive and previously uncharacterized risk architecture. The facilitator is at once a clinical actor, a data handler, a communication broker, and a decision-support agent—functions that in face-to-face care are distributed across a physically co-located team with established protocols and lines of accountability. In the triadic model, these functions converge in a single role operating at geographic remove from the supervising physician, often in resource-limited settings, and without standardized competency frameworks or risk governance protocols. Remote encounters about acute or worrisome symptoms require decision-making under conditions of uncertainty and urgency, and the rapid pace of telehealth’s technological growth creates urgency in identifying safe systems to guide developers and clinicians [15]. Existing patient safety frameworks do not adequately account for this configuration.

### 2.4. Rationale for the Rmftt

The absence of a dedicated risk management framework for triadic telemedicine is consequential rather than merely academic. The populations for whom the triadic model is most relevant—elderly patients, those with cognitive or functional limitations, and residents of underserved areas—carry the greatest clinical risk burden and are the least equipped to self-identify or escalate safety failures during remote consultations. A scoping review of governance strategies for patient safety identified that improvement efforts should combine clinical governance, safety culture, communication, competency, and accountability into an integrated organizational framework [11]. The RMFTT is designed to provide precisely this integration, tailored to the specific structural and operational features of the assisted triadic telemedicine model.

## 3. Methods

### 3.1. Study Design

This study employs a conceptual framework development design, informed by a structured narrative literature review. Conceptual framework development is an established methodology in health systems research for synthesizing heterogeneous bodies of evidence into an integrated, theoretically grounded model when the phenomenon of interest is complex, multidimensional, and lacks an existing organizing structure [16]. This approach is appropriate for the present study, given that risk management in triadic telemedicine has not previously been examined as a unified construct. The five risk domains addressed by the RMFTT draw on distinct bodies of literature—clinical safety, communication science, health law, cybersecurity, and workforce development—which require conceptual integration rather than meta-analytic pooling [16].

The review was conducted and reported in line with the Scale for the Assessment of Narrative Review Articles (SANRA) [17]. A systematic-review methodology such as PRISMA was not adopted, as the aim was conceptual synthesis rather than exhaustive ascertainment of all eligible studies.

The study proceeds through three sequential phases: a structured literature search and mapping phase, a conceptual synthesis phase, and a framework construction phase. Severity classifications represent qualitative expert synthesis derived from the reviewed literature and are intended as heuristic rather than quantitative risk estimates.

### 3.2. Phase 1—Literature Search and Mapping

Literature was identified through PubMed, Scopus, Web of Science, and Google Scholar for English-language publications from January 2015 to June 2026, using domain-specific combinations of telemedicine and risk-relevant terms. The search was not intended to be exhaustive; sources were selected purposively for relevance to the five risk domains and for methodological quality, prioritizing systematic and narrative reviews, implementation studies, and conceptual or framework papers, alongside selected regulatory and governance documents. Where triadic-specific evidence was sparse, particularly for workforce competency, transferable evidence from dyadic telemedicine, nurse-led care, and remote patient monitoring was incorporated and identified as such. Gray literature, including policy documents and regulatory guidance from the World Health Organization and relevant international health agencies, was included where it provided material evidence on risk governance standards not yet reflected in the peer-reviewed literature.

Supplementary Table S1 presents the domain-specific Boolean search strings used across each database and the approximate number of records retrieved per domain before purposive selection.

Following independent domain searches, extracted sources were organized thematically within each domain. Themes were identified inductively from source content and mapped onto the four RMFTT management stages—identification, assessment, mitigation, and monitoring—to guide the framework cell population. Where evidence was transferable from dyadic or nurse-led care contexts rather than triadic-specific, this was noted explicitly in the synthesis.

### 3.3. Phase 2—Conceptual Synthesis

Extracted findings were organized using a structured risk architecture matrix distinguishing four analytical dimensions for each identified risk: risk type (the nature of the potential harm), risk source (the actor or system component from which the risk originates), risk likelihood (assessed qualitatively as high, moderate, or context-dependent), and risk consequence severity (assessed as high, moderate, or low). This four-dimension analytical structure draws on established risk assessment principles in healthcare and digital health governance, including the ISO 14971 standard for the application of risk management to medical devices [18]. ISO 14971 is employed here as a terminological reference only, providing the vocabulary of risk identification, assessment, mitigation, and monitoring; the RMFTT does not constitute a medical device risk management system and does not claim compliance with ISO 14971 requirements. The severity and likelihood ratings represent preliminary author judgements based on the synthesized literature and are intended as heuristic starting points for empirical refinement rather than validated quantitative estimates.

### 3.4. Phase 3—Framework Construction

The RMFTT was constructed as a two-axis conceptual model. The horizontal axis organizes the five risk domains: clinical and diagnostic risk (D1), communication risk (D2), legal and liability risk (D3), data privacy and cybersecurity risk (D4), and workforce competency risk (D5). The vertical axis organizes four risk management stages: identification, assessment, mitigation, and monitoring. The resulting 20-cell matrix constitutes the operational structure of the framework, with each cell populated by literature-derived risk indicators and corresponding management strategies derived from the Phase 2 synthesis. To assist readers in distinguishing the evidentiary basis of individual framework elements, risk indicators and mitigation strategies throughout the domain sections are qualified as follows: items directly supported by a cited source identified in the Phase 1 literature search are denoted as literature-supported; items representing author inference, logical extrapolation from adjacent evidence, or original conceptual contribution are denoted as proposed (plain text, without brackets, to avoid ambiguity with editorial markup conventions).

### 3.5. Scope and Delimitations

The present study is delimited to the triadic model of assisted telemedicine—defined as a three-party remote clinical encounter in which a trained nurse or caregiver facilitates interaction between a patient and a remote physician, as established in the foundational literature on this model [8,9]. The framework is designed to be applicable across health system contexts. This study does not constitute an empirical validation of the RMFTT. The framework is presented as a theoretically grounded conceptual model requiring subsequent empirical testing, and the research program explicitly positions an observational or mixed-methods validation study as the next phase of inquiry. The five risk domains are grounded in universal features of the triadic model that apply across health system contexts, including rural primary care systems in Europe, nurse-facilitated telemedicine programs in low- and middle-income countries, and aged-care facility telemedicine models increasingly prevalent worldwide.

## 4. Results of the Narrative Review

### 4.1. Overview

The narrative review drew on 38 references: 20 providing domain-specific evidence across the five risk domains, and 18 informing the conceptual, methodological, and policy foundations of the framework. Table 1 presents the domain-by-domain references cited, principal source types, and key themes. Across all five domains, the review identified little evidence specific to the triadic model. Evidence gaps were consistent and domain-specific: no primary studies of diagnostic accuracy with a nurse-facilitator intermediary were identified (D1); all communication sources addressed dyadic or face-to-face contexts, making D2 the domain with the highest degree of evidence extrapolation; no legal framework addressing the triadic accountability architecture was found (D3); multi-party data exposure in the triadic configuration was not directly addressed in any identified source (D4); and triadic facilitator-specific competency literature was almost entirely absent (D5)—a scarcity treated as a primary finding of the review rather than merely a limitation.

### 4.2. Domain 1—Clinical and Diagnostic Risk: Review Findings

The evidence for Domain 1 was drawn from three publications. A systematic review of remote diagnosis in telemedicine (2016–2023) mapped the procedures, input variables, and open research issues in remote diagnostic models [19]. The dominant theme across this domain was the conditional nature of diagnostic accuracy in telemedicine—high concordance with in-person diagnosis was consistently demonstrated for conditions relying primarily on patient history and structured questioning, while accuracy declined significantly for conditions requiring physical examination, tactile assessment, or observation of findings not adequately captured by standard video. A systematic review with meta-analysis of telemedicine for musculoskeletal disorders found remote diagnoses highly concordant with in-person diagnoses where physiotherapists served as evaluators, but noted that broader providers including orthopedic surgeons and primary care physicians were underrepresented, limiting generalizability [20].

### 4.3. Domain 2—Communication Risk: Review Findings

Evidence for Domain 2 was drawn from five publications, primarily from the triadic communication literature in face-to-face consultations, the telehealth communication quality literature, and conceptual work on facilitated clinical encounters. A scoping review of patient safety risk in synchronous telehealth, encompassing 15 systematic reviews and 315 studies, identified that patient safety risks in telehealth are systemic, affecting not only healthcare professionals but also the environment and organizational context, with notable gaps in regulatory frameworks and a lack of comprehensive guidelines regarding implementation and monitoring [14]. Communication-specific risk indicators—information filtering, role ambiguity, and patient voice suppression—were identified consistently across the triadic communication literature but have not yet been studied in the telemedicine-specific triadic context.

Physician and patient perspectives add complementary dimensions to this risk landscape. From the physician’s perspective, remote audiovisual consultation introduces structural limitations that compound communication risk: the inability to perform physical examination, reduced access to nonverbal cues, multitasking demands during screen-mediated encounters, and difficulties confirming patient understanding before the consultation ends [21]. These constraints are especially consequential in the triadic setting, where the physician must simultaneously manage a remote clinical encounter and coordinate with a nurse-facilitator whose communicative function may not be clearly delineated. From the patient’s perspective, telemedicine communication barriers centre on difficulties building therapeutic rapport; reliance on technology that many patients—particularly older adults and those with low digital literacy—cannot navigate independently; and reluctance to raise sensitive concerns via video [22]. Patients with low health literacy or language barriers face additional risks of misunderstanding, and may depend on family members or the nurse-facilitator to mediate their communication with the physician, further amplifying the risk of information filtering and patient voice suppression. Together, these bidirectional vulnerabilities underscore the centrality of the nurse-facilitator’s communicative role as both a mitigating resource and a potential source of risk.

### 4.4. Domain 3—Legal and Liability Risk: Review Findings

The evidence for Domain 3 was drawn from four publications, combining empirical legal analysis, regulatory review, and expert commentary. A review of ethical and legal challenges of telemedicine across 24 eligible studies found that legal and ethical issues concerned informed consent and autonomy (cited in 87% of included studies), patient privacy (78%) and confidentiality (57%), data protection and security (74%), and malpractice and professional liability (70%), concluding that ethical and legal issues related to telehealth practice still require standard and specific rules of application [23]. Professional ethics guidance further holds that physicians and organizations providing telemedicine should routinely monitor the telehealth landscape to identify and address adverse consequences as the technology evolves, and should encourage the dissemination of both favorable and unfavorable outcomes [24].

### 4.5. Domain 4—Data Privacy and Cybersecurity Risk: Review Findings

The evidence for Domain 4 was drawn from three publications: platform security evaluations, breach incident analyses, and digital health governance frameworks. A systematic literature review validating telehealth outcome categories for patient safety, covering publications through 2024, identified that integrating telehealth into established care processes presents significant safety evaluation challenges, with existing literature on telehealth outcomes to support evaluation remaining limited [25]. The multi-party data exposure risk specific to the triadic model—arising from the simultaneous presence of three participants on potentially different devices and networks—was not directly addressed in any identified source, though its component risks (unsecured transmission, consent gaps, facilitator data handling errors) were each well-evidenced in the broader telehealth security literature.

### 4.6. Domain 5—Workforce Competency Risk: Review Findings

The evidence for Domain 5 was drawn from five publications, none situated specifically in a nurse-facilitated or assisted telemedicine context. A scoping review of telehealth training and education for allied health professionals concluded that establishing clear training and education standards is crucial, and that ensuring the workforce possesses the required skills, knowledge, and behaviors to provide safe, efficient, and effective telehealth services requires a clear organizational strategy and determination of roles and responsibilities [26]. A modified Delphi study developing telemedicine entrustable professional activities for nurses in long-term care—the closest published analogue to the triadic facilitator role identified in the review—established that developing clear standards of practice is necessary to cater to nurses’ roles and scope in different telemedicine services, with continuing and context-specific education essential to increase nurses’ readiness [27]. A recent scoping review of workforce preparation for telehealth practice, encompassing 31 studies across medicine, nursing, and allied health, confirmed that telehealth services are safe and effective if delivered by appropriately qualified personnel, but found that competence in face-to-face care does not automatically transfer to telehealth delivery and that dedicated training remains essential [28].

## 5. The Rmftt Framework: Domain-by-Domain Construction

This section presents the domain-by-domain construction of the RMFTT, built on the evidence synthesized above. Table 2 summarizes the matrix.

The five-domain synthesis above establishes the RMFTT as a triadic-specific integrative framework. Table 3 compares it with selected telemedicine risk and patient safety frameworks on key structural dimensions, illustrating how the triadic configuration introduces risk architecture not addressed by existing models.

### 5.1. Domain 1—Clinical and Diagnostic Risk

***Identification***

Clinical and diagnostic risk in triadic telemedicine arises primarily from the structural constraints of remote physical assessment and from the mediating role of the nurse-facilitator in the clinical information chain. Remote encounters about acute or worrisome symptoms are time sensitive, requiring decision-making under conditions of uncertainty and urgency, and inadequate telehealth systems can compromise patient safety [15]. Three principal risk indicators are identifiable: limitations of remote physical examination; triage accuracy gaps arising from the nurse-facilitator’s preliminary assessment; and missed clinical signs by an inadequately trained or insufficiently supervised nurse-facilitator. The first two indicators (remote physical examination limitations; triage accuracy gaps) are by multiple systematic reviews identified in Phase 1 [15,19,20]. The third indicator (missed clinical signs by undertrained nurse-facilitator) is proposed, representing a logical inference from the competency literature applied to the triadic context.

***Assessment***

The severity of clinical and diagnostic risk in triadic telemedicine is assessed as high. Misdiagnosis remains one of the most frequently cited patient-safety concerns in telemedicine, particularly when clinical assessment is constrained by the limitations of remote examination [15,19]. Likelihood is assessed as context-dependent, varying with the clinical acuity of the presenting condition, the diagnostic capability of available point-of-care devices, and the competency level of the nurse-facilitator.

***Mitigation***

Three mitigation strategies are indicated: AI-augmented diagnostic support tools integrated into the nurse-facilitator’s workflow; structured triage templates guiding the nurse-facilitator through standardized symptom and vital signs assessment; and clearly defined escalation protocols establishing the threshold at which the telemedicine consultation must be suspended in favor of emergency or in-person care.

Three illustrative failure scenarios clarify the practical relevance of these mitigations. In the first scenario, a nurse-facilitator assessing a patient with atypical chest pain relies on patient-reported symptoms without completing a structured vital signs protocol; the remote physician, unable to auscultate or palpate, does not request a point-of-care ECG, and the patient is discharged with a presumptive musculoskeletal diagnosis before sustaining a myocardial infarction within 12 h. This scenario illustrates the compounding effect of triage accuracy gaps and the absence of escalation protocols. In the second scenario, a nurse-facilitator unfamiliar with paediatric assessment norms underestimates the severity of respiratory distress in a febrile child; the physician, relying on the nurse-facilitator’s verbal description rather than objective measurement, does not escalate to emergency services. This scenario highlights the risk of missed clinical signs by an inadequately supervised nurse-facilitator and underscores the importance of competency-linked scope-of-practice boundaries. In the third scenario, a dermatological image captured under poor lighting conditions leads the physician to classify a lesion as benign; the patient does not receive follow-up, and a subsequent biopsy reveals early-stage melanoma. This scenario illustrates how the limitations of remote physical examination interact with image quality constraints and the absence of structured follow-up protocols to produce diagnostic harm. Each scenario maps directly to an identified mitigation: AI-augmented diagnostic support (Scenario 1), structured triage templates with competency thresholds (Scenario 2), and standardised imaging protocols with mandatory follow-up triggers (Scenario 3).

***Monitoring***

Adverse event reporting systems should capture diagnostic errors, near-misses, and escalation failures attributable to the triadic consultation process. Regular diagnostic accuracy audits comparing nurse-facilitator triage decisions against subsequent physician diagnoses and clinical outcomes provide a continuous quality signal. Outcome benchmarking against comparable dyadic telemedicine or face-to-face care programs enables longitudinal safety assessment.

### 5.2. Domain 2—Communication Risk

***Identification***

Communication risk in triadic telemedicine is structurally distinct from that in dyadic consultations. Triadic communication refers to the presence of a third party in clinical encounters, with the third party assuming a variety of roles, some helpful, some introducing unintended barriers to patient voice and information accuracy [30,31]. Three principal risk indicators are identifiable: role ambiguity; information filtering by the nurse-facilitator; and patient voice suppression, particularly among elderly, cognitively impaired, or lower health literacy patients. Role ambiguity and information filtering by the nurse-facilitator are proposed risk indicators representing original conceptual contributions specific to the triadic model; while information filtering as a general telemedicine risk is proposed by the identified sources [21,22], and its structural amplification through the nurse-facilitator role is an authorial inference. Patient voice suppression among cognitively impaired or low-literacy patients is proposed, extrapolated from the general health communication literature rather than derived from triadic-specific evidence.

From the physician’s perspective, an additional risk indicator is the reduced capacity for real-time assessment of patient distress and non-verbal communication, compounded by the multitasking demands inherent in screen-mediated triadic encounters [21]. From the patient’s perspective, difficulty building rapport and dependence on technology or a family intermediary represent further risk vectors that can suppress accurate symptom reporting and reduce the quality of informed decision-making [22].

***Assessment***

Severity is assessed as moderate to high, given that communication failures in clinical encounters are a well-documented contributor to diagnostic error and adverse outcomes. Key communication challenges in telehealth include the delivery and receipt of information across remote channels, multi-tasking demands on clinicians, difficulties in confirming patient understanding, and managing the follow-up process after the consultation ends, all of which are amplified in the triadic model [32]. Likelihood is assessed as moderate, increasing significantly in consultations involving patients with cognitive impairment, language barriers, or low digital literacy.

***Mitigation***

Three mitigation strategies are indicated: role clarity agreements formally documenting the nurse-facilitator’s communicative function; conversational capacity training addressing the communication pitfalls specific to three-party clinical encounters [33]; and patient voice protocols providing explicit opening invitations for the patient to speak directly to the physician at defined points in the consultation.

***Monitoring***

Consultation recording review enables retrospective assessment of communication quality, including identification of information filtering events and patient voice suppression instances. Patient feedback surveys administered after triadic consultations provide a direct signal of whether patients felt heard and adequately represented. Role conflict incident logs capture cases where ambiguity in the nurse-facilitator’s communicative role contributed to consultation breakdown or patient complaint.

### 5.3. Domain 3—Legal and Liability Risk

***Identification***

The triadic model introduces a distinctive legal risk architecture by distributing clinical responsibility across three actors without a universally established framework for assigning accountability when adverse outcomes occur. In principle, the regime of legal liability for medical malpractice is the same for telemedicine as for traditional physical care; however, the standard of care requires that the overall quality of remote care be at least as good as its comparable physical alternative [34]. Three principal risk indicators are identifiable: accountability gaps arising from the three-actor distribution of clinical responsibility; jurisdictional ambiguity in cross-border or cross-regional deployments; and undefined nurse scope of practice in the triadic role. Accountability gaps and jurisdictional ambiguity are by empirical legal analysis and regulatory review identified in Phase 1 [32,33,34]. Undefined nurse scope in the triadic role specifically is proposed, extrapolated from the general telemedicine scope-of-practice literature to the triadic context.

***Assessment***

Severity is assessed as high, given the potential for adverse patient outcomes and significant organizational liability. Legal and ethical issues in telemedicine—including informed consent, privacy, data protection, and malpractice liability—still require standard and specific rules of application [23]. Likelihood is assessed as system-dependent, varying significantly with the regulatory maturity of the health system in which the triadic model is deployed.

***Mitigation***

Three mitigation strategies are indicated: clear liability frameworks formally documenting institutional accountability for each stage of the triadic consultation; defined nurse scope of care specifying which clinical actions the nurse-facilitator is authorized to perform; and comprehensive informed consent documentation covering all three parties and their respective roles in the consultation.

***Monitoring***

Complaint tracking systems should capture cases involving triadic consultation specifically. Regulatory update alerts ensure institutional protocols are revised whenever national telemedicine legislation changes. Telemedicine implementation continues to be influenced by evolving licensure requirements, reimbursement policies, and regulatory frameworks across jurisdictions, requiring ongoing organizational monitoring and adaptation [3,6]. Periodic malpractice case review provides the most direct monitoring signal.

### 5.4. Domain 4—Data Privacy and Cybersecurity Risk

***Identification***

The triadic model amplifies data privacy and cybersecurity risks by introducing a third access point, the on-site nurse-facilitator, who handles patient data, operates connected diagnostic devices, and transmits biometric measurements through a shared digital session. Three principal risk indicators are identifiable: multi-party data exposure arising from the simultaneous presence of three participants potentially using different devices and network environments; unsecured data transmission; and consent gaps for session recordings specific to the three-party model. Multi-party data exposure and unsecured transmission risks are by platform security and breach incident evidence identified in Phase 1 [25,35,36]. The triadic-specific consent gap indicator is proposed, as no identified source directly addressed three-party recording consent requirements.

Two further risk indicators merit specific recognition in the triadic context. First, identity verification risk arises from the difficulty of confirming patient identity remotely when the consultation is mediated by a nurse-facilitator rather than conducted directly; the physician cannot independently verify that the individual whose data is being discussed is the individual physically present with the facilitator, creating a potential for data attribution errors or deliberate fraud. Second, device and access control risk arises from the use of potentially unmanaged or personally owned devices by nurse-facilitators in community- or home-based deployment settings; where endpoint devices are not enrolled in a mobile device management system, unencrypted data residues, unauthorised application access, and physical theft represent credible threats to patient data integrity. A sociotechnical approach to cybersecurity risk management, addressing not only platform-level controls but also organisational policies and human factors, is indicated by the evidence base [37].

***Assessment***

Severity is assessed as high. Healthcare organizations continue to experience significant cybersecurity threats and data breaches, reflecting persistent vulnerabilities associated with digital health infrastructure, human factors, legacy systems, and interconnected technologies. Recent systematic review evidence suggests that cybersecurity vulnerabilities in healthcare arise from a complex interaction among technological, organizational, and human factors, requiring a sociotechnical approach to risk mitigation [35]. Likelihood is assessed as platform-dependent, varying with the security architecture of the telemedicine system in use.

***Mitigation***

Three mitigation strategies are indicated: GDPR- and HIPAA-compliant platform selection verified through Business Associate Agreements and independent security certification; end-to-end encrypted transmission of all data exchanged during triadic consultations including diagnostic device outputs; and recording consent policies specifically designed for the triadic model covering patient, nurse-facilitator, and physician consent for session recording, data storage, and authorized access.

Two additional mitigation strategies address the identity verification and device security risks identified above. Identity verification should be supported by a standardised patient identification protocol requiring the nurse-facilitator to confirm patient identity against at least two independent identifiers (name and date of birth, or equivalent national identifier) at the outset of each consultation, with confirmation documented in the session record. Device and access control risk should be mitigated through mandatory enrolment of all nurse-facilitator endpoint devices in a centrally managed mobile device management programme, enforcing full-disk encryption, automatic screen lock, remote wipe capability, and application whitelisting. Explicit informed consent for data collection, storage, and sharing should be obtained from patients before initiating triadic consultations and documented in a format compliant with applicable data protection law, with separate consent items for session recording and secondary use of anonymised data. Ewoh et al. (2025) provide a comprehensive sociotechnical framework for cybersecurity governance in connected health settings that maps directly to these control requirements and is recommended as an implementation reference [37].

***Monitoring***

Monitoring of Domain 4 requires breach incident logging; bi-annual platform security audits; consent compliance monitoring; regulatory update tracking; and nurse-facilitator-specific data hygiene checks. Effective cybersecurity governance requires documented risk assessment procedures, breach notification protocols, and maintenance of administrative, physical, and technical safeguards including access controls, encryption, and audit controls [36].

### 5.5. Domain 5—Workforce Competency Risk

***Identification***

Workforce competency risk is the domain most specific to the triadic model and the one with the most limited evidence base, a scarcity that is itself a significant finding, reflecting the absence of established training and competency standards for the nurse-facilitator role. Developing clear standards of practice is necessary to cater to nurses’ roles and scope in different telemedicine services, to ensure continuous uptake and benefit from telemedicine, with continuing and context-specific education essential to increase nurses’ readiness [27]. Three principal risk indicators are identifiable: undertrained nurse-facilitators deployed without adequate preparation; role drift describing the gradual expansion of nurse-facilitator clinical actions beyond formally sanctioned scope; and digital literacy deficits compromising technical performance. Undertrained nurse-facilitator deployment and digital literacy deficits are identified by the telehealth training and competency literature [26,27,28,30,31]. Role drift as a named risk indicator in the triadic facilitator role is proposed, representing an original conceptual contribution not directly addressed in identified sources.

***Assessment***

Severity is assessed as high, given that nurse-facilitator competency underpins the safety performance of all other domains simultaneously. Likelihood is assessed as high based on documented deployment practices: nurse-facilitated telemedicine programs have been rolled out across multiple health systems without the prerequisite competency frameworks, standardized training curricula, or formal scope-of-practice definitions being in place. The risk thus arises not from the absence of standards per se, but from the active deployment of undertrained facilitators into clinical roles whose risk profile has not been systematically characterized or mitigated.

***Mitigation***

Three mitigation strategies are indicated: competency frameworks specifically designed for the triadic nurse-facilitator role drawing on the Entrustable Professional Activities (EPA) methodology; mandatory digital literacy training as a prerequisite for deployment in the triadic role; and supervision protocols establishing regular check-ins between nurse-facilitators and supervising nurses or physicians as a safeguard against role drift. In the United States, the Veterans Affairs Telepresenter Certificate Program—developed in partnership with the University of Florida College of Nursing—trained over 900 healthcare professionals in telepresenting competencies, demonstrating the feasibility and scalability of structured nurse-facilitator training programs [7].

***Monitoring***

Competency re-assessments at defined intervals ensure nurse-facilitator readiness is maintained rather than assumed. A scoping review of telehealth competency evaluation tools identified that reliable, validated instruments for assessing telehealth-specific competencies remain scarce, representing a priority development area for the field [38]. Training completion rate tracking provides an organizational-level signal of program implementation fidelity. Near-miss incident reports are the most sensitive early warning indicator of emerging competency risk and should be encouraged through a non-punitive reporting culture.

### 5.6. Cross-Domain Integration

**Figure 1:**
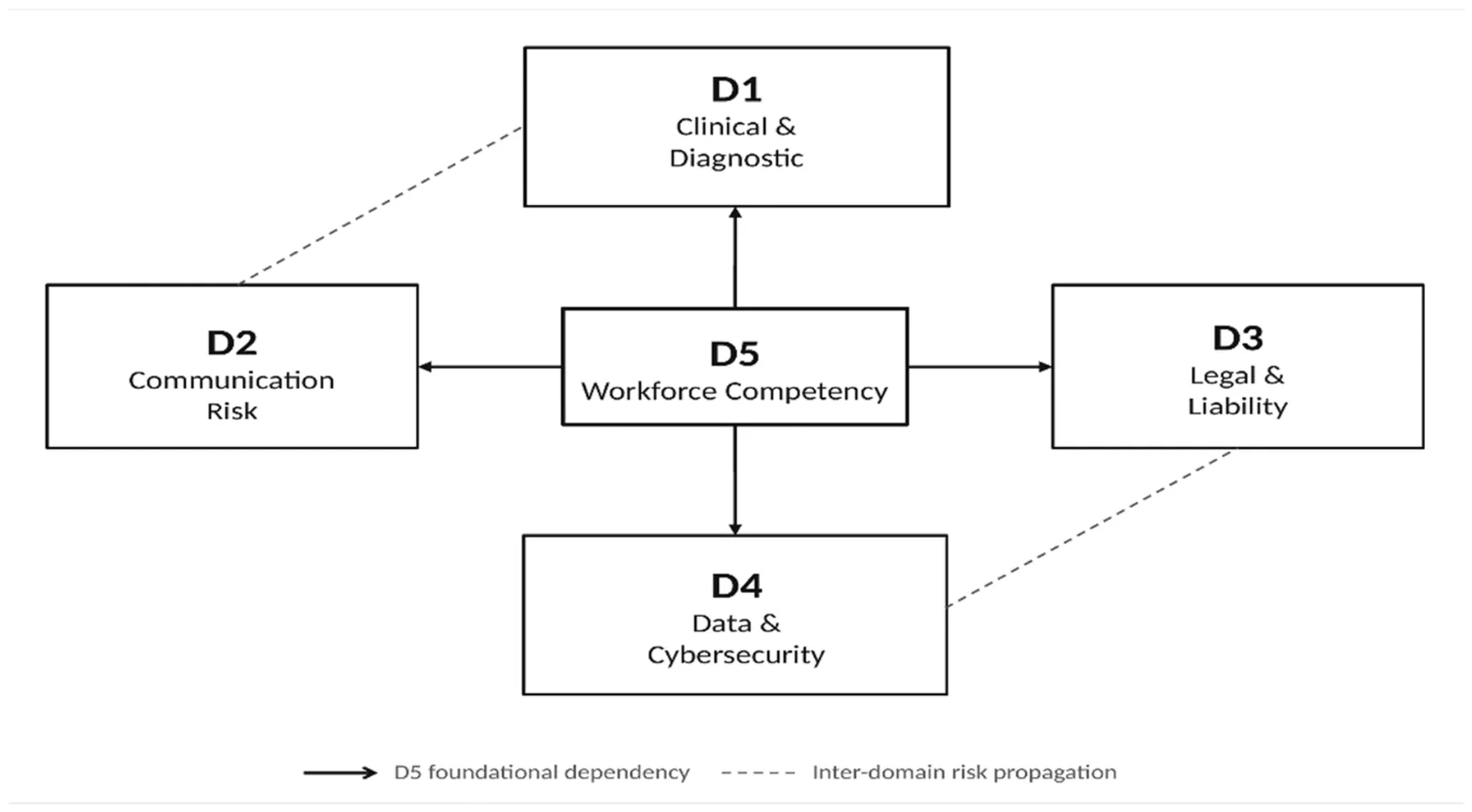
Conceptual dependency structure of the RMFTT: D5 (Workforce competency) functions as the foundational enabling domain, with unidirectional arrows indicating its direct conditioning influence on D1 (Clinical & diagnostic), D2 (Communication), D3 (Legal & liability), and D4 (Data & cybersecurity).

A review of the five domains reveals three cross-cutting structural patterns. First, workforce competency (D5) functions as the foundational enabling domain within the framework: because nurse-facilitator performance shapes the identification, assessment, mitigation, and monitoring of risk across every other domain, inadequate facilitator training simultaneously amplifies clinical diagnostic risk (D1), communication failures (D2), legal liability exposure (D3), and data handling errors (D4) (Figure 1).

This dependency structure suggests that investment in nurse-facilitator competency development delivers risk reduction benefits across the entire framework simultaneously. Similar relationships between workforce capability, system performance, and patient safety outcomes have been described in human factors and socio-technical systems research, where safety is understood as an emergent property of interactions among people, technologies, and organizational processes [39]. Second, communication risk (D2) and clinical risk (D1) are structurally interdependent: information filtering by the nurse-facilitator directly degrades the diagnostic information available to the physician, creating a direct pathway from communication failure to clinical error. Third, legal risk (D3) is activated by failures in any of the other four domains, functioning as a downstream consequence risk rather than an independent upstream cause, a finding with direct implications for institutional governance design, suggesting that legal risk management should be integrated into, rather than siloed from, clinical and operational risk governance.

## 6. Discussion

### 6.1. Principal Findings

This study presents the Risk Management Framework for Triadic Telemedicine (RMFTT)—a structured, domain-specific conceptual framework mapping five risk dimensions against four management stages in the context of nurse-facilitated assisted telemedicine. The framework makes three principal contributions. First, it identifies the triadic model’s distinctive risk architecture—one that differs qualitatively from both face-to-face care and dyadic telemedicine by virtue of the distributed clinical, communicative, legal, and data-handling functions concentrated in the nurse-facilitator role. Second, it demonstrates that these five risk domains are structurally interdependent, with workforce competency (D5) as the foundational enabling domain. Third, it provides an actionable matrix of risk indicators and management strategies directly operationalizable by health system managers, nurse educators, clinical governance teams, and policymakers.

### 6.2. Positioning the Rmftt Within the Existing Literature

The RMFTT builds on and extends a growing body of work on risk and patient safety in telemedicine. Recent work has confirmed that telemedicine risk is multidimensional, with multiple perceived risk categories influencing adoption [40]. Yet this and comparable frameworks have been developed for telemedicine in general, without distinguishing the risk profile of multi-party configurations. The global push to scale up telemedicine services is challenged by complex, multilevel, multifaceted implementation and a lack of consensus on the evidence-based essential building blocks of implementation [3]. The RMFTT addresses this gap by providing a structured, literature-informed set of building blocks specific to the assisted triadic model—a configuration whose risk architecture has not previously been characterized in the literature. The novelty of the RMFTT lies less in the individual mitigation tools than in their systematic organization around risks emerging from the interaction among patient, nurse-facilitator, and physician.

### 6.3. Implications for Practice

For nurse-facilitators, the RMFTT provides an explicit account of the risk functions they carry within the triadic consultation. These functions are currently performed without the benefit of standardized training, formal role definition, or risk governance protocols in most health systems. The identification of role drift as a key workforce competency risk, and the corresponding mitigation strategies centered on competency frameworks and supervision protocols, respond directly to the documented absence of standardized competency frameworks for the nurse-facilitator role identified in the narrative review of Domain 5.

For health system managers and clinical governance teams, the RMFTT’s four-stage structure maps directly onto existing clinical governance frameworks and quality management cycles, facilitating integration rather than requiring the creation of parallel governance structures.

For policymakers, the legal and liability domain (D3) carries particular urgency. The three-party distribution of clinical responsibility in the triadic model creates accountability gaps that current regulatory frameworks in most jurisdictions have not yet addressed. The RMFTT’s articulation of these gaps—accountability ambiguity, undefined nurse scope, and jurisdictional complexity—as identifiable and addressable risk indicators provides a structured basis for regulatory development. The recent enactment of the Uniform Telehealth Act in Washington State (2024) illustrates the kind of regulatory evolution that the RMFTT’s legal domain is designed to support [41].

The applicability of the RMFTT is modulated by the deployment context in which triadic telemedicine is implemented, and practitioners should interpret the framework’s risk assessments in light of local conditions. In rural and geographically remote settings, connectivity constraints represent a structural amplifier of clinical and diagnostic risk (D1): bandwidth limitations degrade video quality, impair the transmission of point-of-care diagnostic device outputs, and increase the likelihood of session interruptions at clinically critical moments. Risk mitigation strategies predicated on high-definition video examination or real-time biometric streaming may require adaptation or substitution in low-bandwidth environments. In low-resource settings more broadly—including community health posts, home-based care programs, and humanitarian response contexts—the assumption of a formally trained and institutionally supported nurse-facilitator may not hold. Where the facilitating role is performed by community health workers, informal caregivers, or volunteers with limited clinical training, the workforce competency domain (D5) severity rating escalates, and the interdependence of D5 with all other domains intensifies correspondingly. Staffing constraints in such settings may also reduce the feasibility of real-time supervision, making asynchronous competency monitoring and structured escalation protocols more important as compensatory controls. Conversely, in well-resourced urban deployment settings with institutional infrastructure, the primary risk amplifiers shift toward regulatory complexity (D3) and data governance (D4), particularly where multiple jurisdictions, institutional systems, and professional licensing frameworks intersect. The RMFTT is designed to accommodate this contextual variation: risk assessors are encouraged to weight domain severity and likelihood ratings according to the specific staffing, infrastructure, regulatory, and connectivity conditions of their deployment environment.

### 6.4. Limitations

Several limitations warrant acknowledgment. First, the RMFTT is a conceptual framework derived from a narrative literature review and has not been empirically validated. Risk indicators, likelihood assessments, and mitigation strategies represent informed conceptual judgements rather than empirically tested propositions. Empirical validation, through observational studies, expert consensus processes, or prospective implementation evaluation, is identified as the next phase of this research program.

Second, the evidence base for several domains, particularly Domain 5 (workforce competency) and aspects of Domain 2 (communication risk specific to the triadic context), is sparse. This scarcity is itself a finding of significance, confirming the novelty of the research area. However, it means that some framework cells draw on transferable evidence from adjacent contexts rather than triadic-specific primary research.

Third, the RMFTT addresses risk management rather than risk quantification. It does not assign numerical probability or severity scores to individual risk indicators, nor provide a composite risk index. Development of validated risk scoring instruments is identified as a priority for future empirical work.

Fourth, as a narrative synthesis, this review is subject to the inherent limitations of that methodology. The purposive, non-exhaustive search strategy, while appropriate for conceptual framework development following the Jabareen [16] procedure, means that the evidence base reflects the authors’ domain expertise and source selection judgements rather than a reproducible algorithmic search. A systematic review or scoping review with pre-registered protocols would provide a more comprehensive and replicable evidence base for subsequent iterations of the RMFTT.

Fifth, the risk of publication bias cannot be excluded. Studies reporting adverse events, system failures, or negative implementation outcomes in telemedicine may be underrepresented in the peer-reviewed literature relative to studies reporting benefits and successes. This asymmetry may lead to underestimation of the frequency or severity of certain risk indicators, particularly in domains where negative outcomes are less likely to be published, such as communication failures and workforce competency gaps. Formal bias assessment tools, such as the ROBINS-I tool for non-randomised studies or the AMSTAR-2 checklist for systematic reviews, were not applied to individual sources, as the study design did not require a formal quality appraisal of included literature. Future empirical validation phases should incorporate structured bias assessment.

Sixth, the RMFTT was constructed without formal expert panel validation. While the framework draws on a substantial body of peer-reviewed literature and is informed by the authors’ clinical and research experience in triadic telemedicine, the absence of a structured expert consensus process—such as a Delphi panel or nominal group technique—means that the domain structure, risk indicators, and mitigation strategies have not been independently appraised by content experts. Expert panel validation is identified as a methodological priority for the next phase of this research program, alongside prospective implementation testing across diverse clinical settings.

### 6.5. Future Research Directions

The RMFTT generates a structured agenda for empirical research. The most immediate priority is the development and validation of a standardized competency assessment tool for the triadic nurse-facilitator role, building on emerging telepresenter competency frameworks. A second priority is a prospective observational study examining communication dynamics and information filtering rates in real-world triadic consultations, directly operationalizing the risk indicators identified in Domain 2. A third priority is a comparative legal analysis of accountability frameworks for triadic telemedicine across health systems. Collectively, these empirical studies would constitute the validation phase of the RMFTT.

### 6.6. Illustrative Vignette: Applying the RMFTT in a Triadic Encounter

The following vignette illustrates the application of the full RMFTT cycle in a representative triadic encounter. A 74-year-old woman with type 2 diabetes, hypertension, and mild cognitive impairment lives in a rural community 90 km from the nearest hospital. Her general practitioner refers her for a specialist nephrology consultation via a nurse-facilitated telemedicine platform. A community nurse with basic telepresenter training is deployed as the on-site facilitator.

Risk identification proceeds across all five domains prior to the encounter. In D1 (Clinical and Diagnostic Risk), the clinical team notes that the patient’s cognitive impairment limits reliable self-reporting of symptoms, and that the nurse-facilitator has no specific training in cognitive assessment or geriatric triage protocols—elevating the risk of triage accuracy gaps. In D2 (Communication Risk), the patient’s limited digital literacy and partial hearing loss create a high likelihood of information loss between the patient, facilitator, and remote nephrologist; the nurse-facilitator has not received structured training in health-literacy-adapted communication. In D3 (Legal and Liability Risk), the scope of the nurse-facilitator’s authority to collect blood and urine samples under direct physician instruction via telemedicine is ambiguous under the applicable regional nursing practice regulations. In D4 (Data Privacy and Cybersecurity Risk), the nurse-facilitator is using a personally owned tablet not enrolled in a mobile device management system, creating an endpoint security gap. In D5 (Workforce Competency Risk), the nurse-facilitator has completed only a four-hour generic telemedicine orientation module and has not been assessed against any triadic-specific competency framework.

Risk assessment assigns high severity to D1, D2, and D5; moderate severity to D3; and high severity to D4 given the unmanaged endpoint device. Likelihood is assessed as high for D2 communication failures and D5 competency gaps, and as context-dependent for D1 given the availability of a point-of-care glucometer and blood pressure cuff.

Mitigation strategies are operationalized before and during the encounter. A structured triage template specific to diabetic nephropathy presentations is completed by the facilitator prior to the video session. The remote nephrologist initiates the consultation with a standardized two-identifier patient verification check conducted through the facilitator. A health-literacy-adapted communication protocol is used, with the facilitator repeating and confirming each clinical question and response. The scope-of-practice ambiguity in D3 is escalated to the clinical governance coordinator before sample collection proceeds; the coordinator confirms that a written physician order transmitted via the secure platform is sufficient legal authorization under the applicable regulations. The endpoint device gap in D4 is flagged for post-encounter remediation; the session proceeds under enhanced session logging as a compensatory control.

Monitoring actions follow the encounter. The facilitator completes a post-session adverse event report noting one near-miss: the patient’s blood pressure cuff reading was transmitted with a 90 s delay due to bandwidth instability, causing a brief period of diagnostic uncertainty resolved by a repeat measurement. This event is logged in the platform’s adverse event reporting system and triggers a review of bandwidth requirements for rural deployment sites. The facilitator’s triage decisions are flagged for inclusion in the next quarterly diagnostic accuracy audit comparing facilitator assessments against specialist diagnoses. The vignette illustrates how the RMFTT’s four-stage cycle—identification, assessment, mitigation, monitoring—operates as an integrated governance tool across all five risk domains within a single clinical encounter, and how the interdependence of the domains (particularly D5 competency underpinning D1 and D2 performance) becomes visible in practice.

## 7. Conclusions

The triadic model of assisted telemedicine, in which a trained nurse or caregiver facilitates the clinical encounter between a patient and a remote physician, represents a promising and increasingly relevant configuration of remote care, particularly for populations whose mobility, cognitive capacity, or technological constraints place standard self-administered telemedicine beyond their reach. Yet the expansion of this model into clinical practice has outpaced the development of structured risk governance. To our knowledge, no dedicated risk management framework has previously been proposed for the triadic telemedicine context. The RMFTT provides the first structured, domain-specific tool for identifying, assessing, mitigating, and monitoring the principal risks inherent to assisted triadic telemedicine.

The RMFTT organizes risk across five domains—clinical and diagnostic, communication, legal and liability, data privacy and cybersecurity, and workforce competency—and maps each domain against four management stages. Its construction reveals that these domains are not independent risk silos but structurally interdependent elements of a coherent risk architecture, with nurse-facilitator workforce competency functioning as the foundational enabling domain whose adequacy determines the safety performance of the model as a whole, and with communication failure constituting a direct upstream pathway to clinical diagnostic risk.

Three conclusions of particular practical and policy significance emerge from this analysis. First, the nurse-facilitator role carries a concentration of risk functions that cannot be managed adequately without purpose-built competency frameworks, supervision protocols, and formal role definitions. Second, the legal and liability architecture of triadic telemedicine remains underdeveloped in most health systems, with accountability gaps and undefined nurse scope of practice representing addressable policy failures rather than inherent characteristics of the model. Third, the data privacy and cybersecurity risk specific to the triadic configuration requires monitoring mechanisms that extend beyond standard dyadic telemedicine compliance frameworks.

The RMFTT is presented as a theoretically grounded conceptual tool requiring empirical validation. Its immediate value lies in providing health system managers, clinical governance teams, nurse educators, and policymakers with a structured, literature-derived basis for designing, auditing, and improving triadic telemedicine programs. Its longer-term value lies in establishing the conceptual architecture for a program of empirical research that will progressively replace conceptual inference with measured evidence.

This study forms part of an ongoing program of research developing the conceptual foundations of triadic telemedicine practice. Building on prior work that established the structural foundations, adoption dynamics, and communicative dimensions of the model [8,9], the RMFTT extends this program to its risk dimension, contributing the conceptual architecture required to equip the field for the empirical phase that follows.

## Figures and Tables

**Table 1:** RMFTT narrative review summary.

Domain	References Cited	Primary Source Types	Key Themes	Evidence Gap
D1 Clinical & diagnostic	3 (14,18,19)	Systematic reviews, meta-analyses	Conditional diagnostic accuracy; triage error rates; physical exam limitations	No triadic-specific diagnostic accuracy studies identified
D2 Communication	5 (12, 25, 26, 27, 28)	Qualitative studies, scoping reviews, conceptual frameworks	Information filtering; role ambiguity; patient voice suppression	No telehealth-specific triadic communication studies identified
D3 Legal & liability	5 (20, 21, 29, 35, 38)	Legal reviews, regulatory analysis, expert commentary	Accountability gaps; informed consent; malpractice risk; scope of practice	No legal framework specific to triadic accountability
D4 Data & cybersecurity	3 (22, 30, 31)	Security breach analyses, compliance reviews, governance frameworks	Multi-party data exposure; GDPR/HIPAA compliance; platform vulnerabilities	No triadic-specific multi-party data security framework
D5 Workforce competency	6 (6, 23, 24, 32, 36, 37)	Scoping reviews, Delphi studies, competency frameworks, certificate programs	Training gaps; role drift; digital literacy deficits; EPA frameworks; telepresenter competencies	Minimal triadic facilitator-specific competency literature
General/Cross-cutting	18	Reviews, risk-management & governance frameworks, policy & methods sources	Risk-management foundations; triadic-model rationale; conceptual & human-factors framing	
Total	40			

Note: Reference numbers in parentheses indicate the sources cited for each domain across its full treatment in the manuscript—the Results synthesis, the corresponding Framework Construction subsection, and the Discussion. Each domain’s findings paragraph summarizes a subset of these sources, with the remainder cited where the framework is developed and discussed.

**Table 2:** RMFTT summary matrix: five risk domains mapped against four management stages.

Domain	Identification	Assessment	Mitigation	Monitoring
D1 Clinical & diagnostic	Remote exam limits; triage gaps; missed signs	Severity: high; Source: nurse + system	AI decision support; structured templates; escalation protocols	Adverse event logs; diagnostic audits; outcome benchmarks
D2 Communication	Role ambiguity; information filtering; patient voice suppression	Severity: moderate-high; Source: all three actors	Role clarity agreements; conversational capacity training; voice protocols	Consultation recording review; patient surveys; role conflict logs
D3 Legal & liability	Accountability gaps; jurisdictional ambiguity; undefined nurse scope	Severity: high; Source: org + policy	Liability frameworks; defined nurse scope; informed consent docs	Complaint tracking; regulatory alerts; malpractice case review
D4 Data & cybersecurity	Multi-party data exposure; unsecured transmission; consent gaps	Severity: high; Source: tech + user	GDPR/HIPAA compliance; encryption; recording consent policy	Breach incident logs; platform audits; consent compliance checks
D5 Workforce competency	Undertrained facilitators; role drift; digital literacy deficits	Severity: high; Source: facilitator	Competency frameworks; mandatory digital training; supervision protocols	Competency re-assessments; training completion rates; near-miss reports

**Table 3:** Comparison of RMFTT with existing telemedicine risk and patient safety frameworks.

Framework	Scope	Risk Domains	Triadic/Multi-Party	Evidence Base	Management Stages	Empirically Validated
Moonsamy & Ahmed 2023 [5]	General e-health	Data breaches, cyberattacks, software malfunction, privacy violations, legal/ethical implications	No	Literature-based	Identify -> Assess -> Mitigate -> Review	No
Edmunds et al. 2017 [6]	Telehealth (policy)	Regulatory environment, delivery system infrastructure, outcomes measurement	No (dyadic)	Policy review; expert consensus	Not specified	No
De Micco et al. 2022 [29]	Telemedicine (COVID-era)	Human factors, patient-physician communication, informed consent, patient identification, structural/technological factors	Partial (caregiver cited)	Narrative review + ethics analysis	Risk profile; 13-point position statement	No
Ferrara et al. 2024 [13]	AI-enabled healthcare (broad)	Algorithmic bias, adverse events from AI, medication errors, incident reporting	No	Systematic review (AI safety)	Not specified	No
Oliveira et al. 2025 [14]	Synchronous telehealth	Patient experience, medication safety, communication, training & education, patient identification	No (dyadic)	Scoping review (15 SRs, 315 studies)	Not specified	No (descriptive)
ISO 14971:2019 [18]	Medical devices (incl. software/AI)	Hazard identification, probability, severity, residual risk control	No	International normative standard	Identify -> Analyse -> Evaluate -> Control -> Review	Yes (normative)
RMFTT (present study)	Assisted/triadic telemedicine	Clinical/diagnostic (D1), communication (D2), legal/liability (D3), data privacy & cybersecurity (D4), workforce competency (D5)	Yes (patient-nurse-facilitator-remote physician)	Narrative synthesis (36 sources, 2015–2025)	Identify -> Assess -> Mitigate -> Monitor	Conceptual (pending validation)

## Data Availability

No human-participant dataset was generated or analyzed. The framework was developed from published literature and policy documents. The source-mapping and framework extraction table is available from the corresponding author upon reasonable request.

## Supplementary Materials

The following supporting information can be downloaded at: https://www.mdpi.com/article/10.3390/healthcare14213747/s1. Supplementary Table S1. Domain-Specific Boolean Search Strings and Approximate Record Counts.
