# Supplementary material for: A Risk Management Framework for Triadic Telemedicine (RMFTT): A Conceptual Development Study
Source: Healthcare (Basel). 2026 Nov 3;14(21):3747. doi: 10.3390/healthcare14213747 (PMC13763339; doi:10.3390/healthcare14213747)
Supplement: Supplementary file 1 [file healthcare-14-03747-s001.zip › healthcare-4626183-supplementary.pdf]

Supplementary Table S1. Domain-Specific Boolean Search Strings and Approximate Record Counts.

Risk Domain	Search String (Boolean)	Databases	Approx. Records Retrieved
D1: Clinical and Diagnostic Risk	("telemedicine" OR "telehealth" OR "remote consultation") AND ("clinical risk" OR "diagnostic accuracy" OR "misdiagnosis" OR "physical examination" OR "point-of-care" OR "patient safety") AND ("risk management" OR "framework" OR "systematic review")	PubMed, Scopus, WoS	~320
D2: Communication Risk	("telemedicine" OR "telehealth" OR "video consultation") AND ("communication" OR "language barrier" OR "health literacy" OR "patient-physician communication" OR "misunderstanding") AND ("risk" OR "safety" OR "quality")	PubMed, Scopus, WoS, GS	~410
D3: Legal and Liability Risk	("telemedicine" OR "telehealth" OR "digital health") AND ("legal" OR "liability" OR "malpractice" OR "informed consent" OR "licensure" OR "cross-border" OR "jurisdiction") AND ("risk" OR "framework" OR "governance")	PubMed, Scopus, GS	~285
D4: Data Privacy and Cybersecurity Risk	("telemedicine" OR "telehealth" OR "eHealth" OR "mHealth") AND ("cybersecurity" OR "data privacy" OR "data breach" OR "GDPR" OR "HIPAA" OR "encryption" OR "identity verification" OR "access control") AND ("risk" OR "patient safety")	PubMed, Scopus, WoS	~370
D5: Workforce Competency Risk	("telemedicine" OR "telehealth") AND ("nurse" OR "nurse-facilitator" OR "telepresenter" OR "workforce") AND ("competency" OR "training" OR "burnout" OR "digital literacy" OR "scope of practice") AND ("risk" OR "safety")	PubMed, Scopus, WoS, GS	~240
Triadic / Assisted Telemedicine (cross-domain)	("triadic telemedicine" OR "assisted telemedicine" OR "nurse-facilitated telemedicine" OR "telepresenter" OR "patient-site presenter") AND ("risk" OR "safety" OR "framework")	All 4 databases	~45

GS = Google Scholar; WoS = Web of Science. Record counts are approximate and reflect initial retrieval before purposive selection.
Search period: January 2015 – June 2026. Language restriction: English.
